# Knockdown of the *GABARAP* ortholog *Atg8a* elicits deficits in learning and promotes obsessive behaviors in *Drosophila melanogaster*

**DOI:** 10.17912/micropub.biology.001116

**Published:** 2024-05-30

**Authors:** Theodore Hatfield, Seth Johnson

**Affiliations:** 1 Department of Biology, Simmons University, Boston, Massachusetts, United States

## Abstract

In humans, trafficking of the GABA(A) receptor by GABARAP can lead to obsessive behaviors and learning deficits often in seen in neurological disorders such as Tourette’s Syndrome. We find that in *Drosophila melanogaster* , *Atg8a* , the ortholog of the human *GABARAP* gene, is necessary in the nervous system for learning and suppression of excessive grooming. These results suggest that knocking down *Atg8a* in neurons of *Drosophila* produces a phenotype similar to that seen in human patients, potentially allowing for use of an *Atg8a* knockdown background as a suitable invertebrate model for related neurological conditions.

**Figure 1. Knockdown of Atg8a increases obsessive grooming and reduces larval learning:**
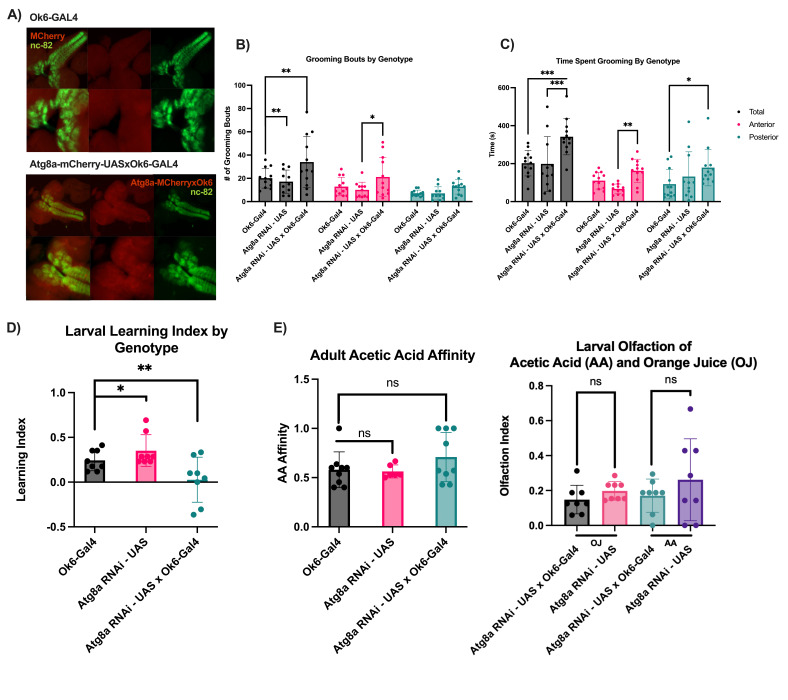
(A) *Ok6-Gal4 shows partial CNS expression.* 3 ^rd^ instar larvae expressing *Ok6-Gal4* with *UAS-mCherry* or *Ok6-Gal4* alone were dissected and brains were removed. Dissected larval brains were fixed and stained with antibody against Brp as a CNS marker. CNS expression of *Ok6-Gal4* driver was noted as colocalization of mCherry and Brp in clearly defined regions absent from the *Ok6-Gal4* control. (B) *Number of grooming bouts were greater in Atg8a knockdown flies* <1 week old adult flies were observed in a viewing chamber and then analyzed for number of anterior and posterior grooming bouts over a 15 minute period. Number of total and anterior, but not posterior grooming bouts were significantly increased compared to controls, suggesting Atg8a normally suppresses initiation of grooming. *p=0.039, **p=0.001 ( *Atg8aRNAi* vs. *Atg8aRNAi* x *Ok6 Gal4* ), **p=0.007 ( *Ok6 Gal4* vs. *Atg8aRNAi* x *Ok6 Gal4* ) (C) *Time spent grooming was increased in Atg8a knockdown flies* <1 week old adults were observed in a viewing chamber for 15 minutes and the duration of anterior and posterior grooming bouts was quantified. Grooming bout duration was significantly increased as a total and when broken into anterior and posterior modules. *p=0.036. **p=0.022, ***p=0.0004 ( *Atg8aRNAi* vs. *Atg8aRNAi* x *Ok6 Gal4* ), ***p=0.0005 ( *Ok6 Gal4* vs. *Atg8aRNAi* x *Ok6 Gal4* ) (D) *Neuronal knockdown of atg8a decreases learning index* . For this and all subsequent experiments *Ok6-Gal4* and *UAS-Atg8aRNAi* flies were crossed and compared to control strains with *Ok6* and *Atg8a RNAi* alone. To ensure reproducibility, three separate lines for *Atg8a RNAi* were used for all experiments, and all showed similar results. Odor taste learning assays were performed on wandering third instar larvae according to methods published in Ueoka et al. (see methods). Learning index was calculated which ranges from 0 (no learning) to +1 (perfect associative learning) and -1 (perfect inverse learning). RNAi knockdown of *Atg8a* resulted in a reduced learning index compared to controls suggesting it is necessary for proper larval learning. *p=0.044, **p=0.001 (E) *Overall olfaction in adults and larvae was not significantly affected by Atg8a knockdown* . To determine whether *Atg8a* knockdown flies had any defects in olfaction, <1 week old adults were starved and placed in a T-maze allowing flies to choose between acetic acid or a water control. Knockdowns for Atg8a showed no significant difference in preference for acetic acid, which they are normally attracted to. To determine if larval olfaction was altered, larvae were also tested to determine if olfaction to a moderate (orange) and strong (acetic acid) scents were significantly different between *Atg8a* KD and controls

## Description

Neurological disorders such as Tourette’s Syndrome can cause involuntary movements and vocalizations known as tics. The condition is fairly common with prevalence of 0.77% of all children, but is more common in males with a prevalence of 1.06% (Knight et al., 2012) . It is often accompanied by learning delays, anxiety, behavioral problems, sleep disorders or other comorbidities– often attention deficit hyperactivity disorder (ADHD), obsessive compulsive disorder (OCD) and/or autism spectrum disorder (ASD) (Hsu et. al, 2021, Burd et. al. 1992) . Though the pathophysiology of TS is poorly understood, abnormal amounts of neurotransmitters or hyperinnervation is likely to blame with the majority of research specifically related to the release or reuptake of dopamine (Buse et al., 2013; Hienert et al., 2018) . In addition to dopamine, a role in GABA and glutamate has also been proposed, with several studies finding reduced concentrations of GABA in children with TS (Freed et al., 2016; Puts et al., 2015) . Trafficking of the GABA _A_ receptor to the cell surface is controlled by GABA­ _A_ receptor associated protein (GABARAP), which itself has been negatively correlated with tic severity in TS patients (Hsu et al., 2021; Tian et al., 2011) .

To better understand the role of GABARAP in TS, we utilized the *UAS-GAL4* system commonly used in *Drosophila* (Southall et al., 2008) to overexpress RNAi against the *Drosophila* homolog of GABARAP, *Atg8a* , in the nervous system to reduce *Atg8a* gene expression. To minimize interference with sensation, we expressed RNAi against *Atg8a* using the *Ok6-Gal4* driver, which primarily expresses in motor neurons with some expression in the central nervous system but not sensory neurons ( Figure 1A ), (Lembke et al., 2017; Sanyal, 2009) . To confirm expression in CNS, we expressed *UAS-mCherry* using *Ok6-Gal4* and observed colocalization with Brp (nc-82), a marker for larval CNS expression ( Figure 1A ). Human patients with TS often experience repetitive, obsessive behaviors and learning deficits (Müller N. 2007). To determine if knockdown (KD) of *Atg8a* resulted in TS-like behaviors in flies, we assessed learning in larva and grooming behaviors in adults. Grooming behavior involves repetitive front and hind limb movements that are primarily used to clean bits of foreign material off of the fly’s legs and body (Sachs, 1988; Szebenyi, 1969) . This behavior is modular with distinct neurological programs each responsible for grooming behavior in different anatomical regions and suppressing grooming programs in other regions (Seeds et al., 2014) . *Drosophila* models of other neurological disorders including fragile X syndrome and autism spectrum disorder have observed effects on grooming, demonstrating the usefulness of observing this behavior in determining neuratypical genetic backgrounds (Andrew et al., 2021) .

Upon KD of *Atg8a* using the *OK6-Gal4* driver of *Atg8a* RNAi, we observed an overall increase in grooming bout behaviors relative to individual *Gal4* and *UAS* controls, suggesting Atg8a normally plays a suppressive role in neurological grooming programs ( Figure 1B ). To determine if this role was biased toward programs controlling anterior or posterior grooming bouts, we specifically quantified each behavior separately. This revealed a significant increase in anterior, but not posterior grooming upon *Atg8a* KD, suggesting that Atg8a is specifically responsible for suppressing the neural program responsible for cleaning the head and not the abdomen. This may suggest a role for Atg8a in a *Drosophila* model separate from a ASD/FXS model seen in *dFMR1* mutants which specifically resulted in an increase in posterior grooming (Andrew et al., 2021) . We also quantified the total time spent grooming because persistence of a grooming bout may be controlled separately from its initiation. We observed significant increases in time spent grooming overall which was also seen in anterior and posterior grooming behaviors ( Figure 1C ). Taken together these data reveal a clear suppressive role for Atg8a in controlling grooming behaviors which may be dependent on anatomical position on the adult fly.

Human patients with TS often exhibit comorbid behaviors including deficits in learning and memory (Gorman et al., 2010) . To determine if these deficits could be replicated in *Drosophila* , we utilized a larval learning assay commonly used to assess learning deficits in other fly neurological disorder models (Ueoka et al., 2019) . To do this, 3 ^rd^ -instar larvae were collected and split into two groups. One group of larvae were exposed to n-amyl acetate in the presence of a sucrose reward and then exposed to 1-octanol without the reward. The second group of larvae underwent a similar process, except the reward was associated with 1-octanol instead of n-amyl acetate. This training was repeated three times, and then the trained groups were transferred to an agar plate, with the two odorants on opposite sides. The larvae were then placed in the center and after twenty minutes their displacement was observed. From this, the learning index of each group was calculated (see methods). Results from this assay indicated a significant decrease in learning index compared to *UAS* and *Gal4* controls, suggesting a necessity for Atg8a in the nervous system in learning and memory ( Figure 1D ). Given that larvae depend on olfaction to guide them to the odor associated with the reward, we also tested whether KD of *Atg8a* affected olfaction in general. We found that *Atg8a* KD larvae did not significantly affect their ability to sense and move toward the scent of oranges, an odor for which flies have an intermediate level of general attraction ( Figure 1E ) (Dweck et al., 2018) . In addition, adult flies had no trouble in using olfaction to migrate to the side of a T-maze containing an attractive odorant (acetic acid), demonstrating that adult flies as well as larvae were not deficient in overall olfaction or mobility ( Figure 1E )

Our goal with this study was to determine if, in flies, knockdown of a gene suspected to be involved in human TS elicited obsessive behavior and learning in *Drosophila* . Here, we show that *Atg8a* , whose human homolog *GABARAP* is negatively correlated with tic severity in human TS patients, is necessary for suppression of grooming behaviors in adult flies and proper learning and memory in 3 ^rd^ instar larvae. These results suggest that a genetic background in *Drosophila* where *Atg8a* is knocked down could serve as a model for further study of TS in a model organism. However, since TS in humans is polygenic, we are not suggesting that *Atg8a* alone is causative. Additionally, human patients display varied behaviors beyond what we have observed here, and it is currently unclear how *Atg8a* could be connected to these observed phenotypes. *Atg8a* has roles related to autophagy as well as trafficking of the GABA _A_ receptor and therefore knockdown could contribute to these behavioral phenotypes through either or both mechanisms. It is also interesting that these phenotypes arose by knocking down *Atg8a* primarily in motor neurons. Independent of *Atg8a* knockdown in the CNS, these results could suggest a role for autophagy or GABA _A_ receptor trafficking in motor neurons for control of grooming and learning behaviors.

## Methods

**Fly maintenance and handling**

All flies were maintained on a cornmeal medium (Fly Food J, LabExpress) at 25°C with a 12h light/dark cycle. *Atg8a* RNAi and *Ok6 Gal4* stocks were purchased from the Bloomington Drosophila Stock Center (BDSC). Flies were anesthetized using CO _2_ , except for when preparing for the Grooming assay when cold anesthetic was instead used.

**Grooming assay**

12 adult flies (6 male, 6 female) were selected and individually placed in observation chambers over grid paper. The specimens are then filmed using the PiSpy program for 15 minutes using a Raspberry pi camera and a tripod. Filming of the flies occurred once every 30 minutes after the start of the 24 hour light cycle (12 hours light : 12 hours dark) and footage was analyzed in the Elan 6.5 software. The number and length of both anterior and posterior grooming bouts, defined as a period of time without a 2 second break between behaviors, was recorded and then analyzed for the following factors: the overall number of bouts and the overall percentage of time spent grooming. Additional data was collected on frequency and time spent engaging in the following behaviors: walking, standing still, falling, and sleeping. The data collected summarizes the behavior of 36 observed flies over 3 genotypes ( *OK6* and *UAS-Atg8a* RNAi separate controls and *Ok6 x UAS-Atg8a* RNAi knockdown)

**Odor Taste Learning Assay** (from Ueoka *et. al* )

Larvae from each genotype were divided into two groups. One group of larvae were first exposed to n-amyl acetate (diluted by liquid paraffin by a factor of 1:50) on a 0.2M sucrose fortified 2% agarose reward dish for ten minutes. Following this, the same group of larvae were exposed to 1-octanol without a reward for an equal amount of time. A second group of larvae were exposed in the same way, but with the odorants and sucrose reversed. This training process is then repeated twice after which each individual group of about thirty were transferred to an agar plate without sucrose with both odorants on opposite sides of the plate. The larvae are placed in the center, and then after three minutes the placement of the larvae were observed. The affinity the groups had for each odorant is then calculated using the following formulas: a = number of larvae on n-amyl acetate side, t = number of larvae on 1-octanol side, (t - a)/total number of larvae = 1-octanol preference. From these calculated preferences, the learning index (LI) can be calculated using the following formulas: n-amyl acetate trained OCT preference = NA, 1-octanol trained OCT preference = OC

LI = (OC - NA) / 2

**Olfaction**

The larval olfactory assay was performed by randomly selecting a group of larvae and placing them on an 1% agarose dish between two equidistant filter paper pieces containing an odorant. Two odorants were tested in this experiment, one being a strong odor (10% acetic acid) and another being more subdued (orange juice). Larvae were placed in the center of the two papers, where the odorants are of equal intensity, and then after five minutes the placement of the larvae are observed. A 2 cm radius was established around each filter paper and if a larva was within those 2cm they are each larva was assigned a number within a binary system representing if they were in this olfactory zone (OZ) or not. The number one was a score given to those larvae that were within the 2cm olfactory zone towards and zero was the score given to those that were not. The individual values of each test group were then assigned to 8 random groups for analysis. The olfactory index the groups had for each odorant was then calculated using the following formula:

OI = (total larvae within OZ)/(total larvae within OZ)+(total larvae outside OZ)

**Immunohistochemistry and Imaging**

To obtain CNS images 3rd instar larvae were dissected, leaving CNS attached to the cuticle. Samples were then fixed in PBS (1x) containing 2% para-formaldehyde for 20 min with rocking. The tissues were then removed from the fixative and briefly washed 3 times with PBS (1x). The CNS was then removed from the cuticle along with as much surrounding tissue as possible and the sample placed in a 1.5mL Eppendorf tube containing a 1:200 dilution of primary nc-82 (mouse) antibody. The tissue was incubated for 2 days at 4 ^o^ C and then undergoes three 10 minute washes at room temperature and a fourth overnight wash at 4 ^o^ C. After the overnight wash, three more 10 minute washes at room temperature are performed and the sample is then incubated with secondary antibody at a 1:200 dilution for 2 days. The same wash cycle as used with the primary antibody is performed, and the whole samples were then mounted on the slide. Images were collected under 488 nm and 561 nm wavelengths at 1000 ms and 2100 ms of exposure respectively. Processing of images involved orthogonal projection of the z-stack taken of the brain tissue.

**T-Maze**

For this assay, a group of ~30 flies were starved for at least 12 hours to promote exploration during the test. Starved flies were then transferred to a small loading vial connected to a t-shaped plastic junction. This connection was made by a transfer pipette that had been cut at both ends and fastened to the loading vial and junction. Attached to the other two ends of the junction are two similarly fastened to “trap” vials. These were also connected by transfer pipettes that had been cut at the bulb, but the thin tip on the end of the pipette that connects into the “trap” vial remained uncut to prevent backward movement of the flies once they entered this vial. One of these vials contained a filter paper dipped in the olfactory stimulus 10% acetic acid, while the other was empty. Flies were then left for 24 hours at 25 ^o^ C in the t-maze apparatus. After this time, the number of flies in each vial will be observed and quantified. The acetic acid preference is calculated with the following equation:

(number of flies in the acetic acid vial - number of flies in the blank vial)/total number of flies = acetic acid preference

**Statistical Analysis**

For olfactory and learning data significance was calculated using the Student’s T-test. For Grooming data significance was calculated using a two-way ANOVA test. All analysis was done using GraphPad Prism version 10.1.0 for iOS, GraphPad Software, Boston, Massachusetts USA, www.graphpad.com

## Reagents

Drosophila stocks used:

*Ok6-Gal4* (Bloomington Stock Center, 64199)

*UAS-Atg8a RNAi* (Bloomington Stock Center, 34340)

UAS-Atg8a-mCherry (Bloomington Stock Center, 37750)

Antibodies Used:

Bruchpilot (Brp, Nc82)

The monoclonal antibody was obtained from the Developmental Studies Hybridoma Bank, created by the NICHD of the NIH and maintained at The University of Iowa, Department of Biology, Iowa City, IA 52242
